# A Taiwanese population-based study on the association between chronic tonsillitis and tonsil cancer

**DOI:** 10.18632/oncotarget.24262

**Published:** 2018-01-17

**Authors:** Shih-Han Hung, Li-Ting Kao, Chung-Chien Huang, Ben-Chang Shia, Herng-Ching Lin

**Affiliations:** ^1^Department of Otolaryngology, Taipei Medical University Hospital, Taipei, Taiwan; ^2^Department of Otolaryngology, School of Medicine, College of Medicine, Taipei Medical University, Taipei, Taiwan; ^3^Graduate Institute of Life Science, National Defense Medical Center, Taipei, Taiwan; ^4^School of Health Care Administration, Taipei Medical University, Taipei, Taiwan; ^5^Big Data Research Center, Taipei Medical University, Taipei, Taiwan; ^6^Sleep Research Center, Taipei Medical University Hospital, Taipei, Taiwan

**Keywords:** chronic tonsillitis, tonsil cancer, epidemiology

## Abstract

Although it is known that inflammatory processes elevate the risk of cancer, to date the association between chronic tonsillitis and tonsil cancer remains unknown. This study aimed to evaluate the association of chronic tonsillitis with tonsil cancer based on a population-based database in Taiwan. We retrieved data for this study from the Longitudinal Health Insurance Database 2005. This case-control study included 489 patients with tonsil cancer and 2445 matched controls. We used conditional logistic regression analyses to calculate the odds ratio (OR) and corresponding 95% confidence interval (CI) for having been previously diagnosed with chronic tonsillitis between patients with tonsil cancer and the controls. We found that of the 2934 sampled patients, 22 (0.75%) had received a diagnosis of chronic tonsillitis. A Chi-squared test further revealed that there was a significant difference in the prevalences of prior chronic tonsillitis between tonsil cancer patients and controls (2.45% vs. 0.41%, *p*<0.001). The conditional logistic regression suggested that after adjusting for hypertension, diabetes, hyperlipidemia, obesity, tobacco use disorder, and alcohol abuse/alcohol dependency syndrome, the OR of having previously been diagnosed with chronic tonsillitis for patients with tonsil cancer was 8.07 (95% CI: 3.32∼19.64; *p*<0.001) compared to controls. It is also noteworthy that alcohol abuse/alcohol dependency syndrome was significantly associated with TC (adjusted OR=9.88). We demonstrated that patients with chronic tonsillitis were more likely to have tonsil cancer, and our findings support tonsillitis as a possible risk factor for tonsil cancer.

## INTRODUCTION

The global incidence of lip, oral cavity, and pharyngeal cancers was reported to be 529,500 in 2012, corresponding to 3.8% of all cancer cases, and it is predicted to rise by 62% to 856,000 cases by 2035 because of changes in demographics [1]. The incidence of oropharyngeal cancers, including tonsil cancer (TC), is increasing in the United States, while the incidence of oral cavity cancers has declined [2, 3].

This change in disease patterns has generated interest in understanding the etiology and pathogenesis of the development of oropharyngeal cancers. Most of the emphasis has been on the human papillomavirus (HPV), and it was found that increases in the population-level incidence of oropharyngeal cancers in the United States since 1984 were caused by HPV infection [4].

On the other hand, tonsillitis, a very common disease, is seldom regarded as a significant risk factor for oropharyngeal carcinomas. The relationship between inflammation and carcinogenesis is well documented [5, 6]. The phenomenon is also supported by our previous studies regarding associations between the development of nasopharyngeal carcinoma and surrounding inflammatory conditions [7, 8].

Although it is known that inflammatory processes elevate the risk of cancer, the association between chronic tonsillitis and TC remains unknown to date [9, 10]. The purpose of this study was to evaluate the association of chronic tonsillitis with TC based on a population-based database in Taiwan.

## RESULTS

We found that the mean age of the 2934 sampled patients was 55.4 ± 12.7 years. Table 1 shows that after matching for sex, age, monthly income, geographical location, urbanization level of the patient’s residence, and year of the index date, we observed no significant differences in hypertension, diabetes, hyperlipidemia, obesity, or tobacco use disorder between patients with TC and controls. However, patients with TC were more likely to have a diagnosis of alcohol abuse/alcohol dependency syndrome than did the controls (*p*<0.001).

**Table 1:** Demographic characteristics of patients with tonsil cancer and controls in Taiwan (N=2934)

Variable	Patients with tonsil cancer (*n*=489)		Controls (*n*=2445)		*p* value
	Total no.	Percent (%)	Total no.	Percent (%)	
Age (years), mean (SD)	55.4 (12.7)		55.4 (12.7)		>0.999
Males	408	83.4	2040	83.4	>0.999
Urbanization level					>0.999
1 (most urbanized)	104	21.3	520	21.3	
2	133	27.2	665	27.2	
3	89	18.2	445	18.2	
4	82	16.8	410	16.8	
5 (least urbanized)	81	16.6	405	16.6	
Monthly income					>0.999
≤NT15,840	191	39.0	955	39.0	
NT$15,841∼25,000	220	45.0	1100	45.0	
≥NT$25,001	78	16.0	390	16.0	
Geographic region					>0.999
Northern	158	32.3	790	32.3	
Central	167	34.2	835	34.2	
Southern	144	29.4	720	29.4	
Eastern	20	4.1	100	4.1	
Obesity	2	0.4	28	1.2	0.140
Hypertension	158	32.3	797	32.6	0.902
Diabetes mellitus	80	16.4	368	15.1	0.463
Hyperlipidemia	102	20.9	598	24.5	0.088
Tobacco use disorder	18	3.7	60	2.5	0.124
Alcohol abuse/alcohol dependence syndrome	19	3.7	9	0.4	<0.001

SD, standard deviation.

Note: The average exchange rate in 2008 was US$1.00≈New Taiwan (NT)$29.

The prevalence of prior chronic tonsillitis between patients with TC and controls is presented in Table 2. Of the 2934 sampled patients, 22 (0.75%) had received a diagnosis of chronic tonsillitis before the index date. A Chi-squared test further revealed that there was a significant difference in the prevalence of prior chronic tonsillitis between TC patients and controls (2.45% vs. 0.41%, *p*<0.001).

**Table 2:** Prevalence and odds ratios (ORs) for prior chronic tonsillitis among sampled subjects

Presence of prior chronic tonsillitis	Total (*N=*2934)		Patients with tonsil cancer (*N*=489)		Controls (*N*=2445)	
	*n*, Percent (%)		*n*, Percent (%)		*n*, Percent (%)	
Yes	22	0.75	12	2.45	10	0.41
OR (95% CI)	--		6.17^***^ (2.65∼14.39)		1.00	

Note: The OR was calculated by a conditional logistic regression which was conditioned on sex, age, monthly income, geographical location, urbanization level of the patient’s residence, and year of the index date; ^***^ p<0.001.

CI, confidence interval.

The ORs and corresponding 95% CIs for having been previously diagnosed with chronic tonsillitis between patients with TC and controls are also shown in Table 2. The conditional logistic regression (conditioned on sex, age, monthly income, geographical location, urbanization level of the patient’s residence, and year of the index date) suggested that the OR of prior chronic tonsillitis for patients with TC was 6.17 (95% CI: 2.65∼14.39, *p*<0.001) compared to controls.

Furthermore, we found that after adjusting for hypertension, diabetes, hyperlipidemia, obesity, tobacco use disorder, and alcohol abuse/alcohol dependency syndrome, the OR of having previously been diagnosed with chronic tonsillitis diagnosis for patients with TC was 8.07 (95% CI: 3.32∼19.64; *p*<0.001) compared to controls (Table 3). Also, it is noteworthy that alcohol abuse/alcohol dependency syndrome was significantly associated with TC (adjusted OR=9.88, 95% CI=4.34∼22.49, *p*<0.001).

**Table 3:** Covariate-adjusted odds ratios for tonsil cancer among the sampled patients (N=2934)

Variable	Tonsil cancer occurrence		
	Odds ratio	95% CI	*p* value
Chronic tonsillitis	8.07	3.32∼19.64	<0.001
Obesity	0.33	0.08∼1.40	0.132
Hypertension	1.00	0.77∼1.29	0.975
Diabetes mellitus	1.33	0.98∼1.81	0.069
Hyperlipidemia	0.77	0.58∼1.02	0.064
Tobacco use disorder	1.31	0.74∼2.34	0.356
Alcohol abuse/alcohol dependence syndrome	9.88	4.34∼22.49	<0.001

Note: The odds ratios were all derived from the same conditional logistic regression which was conditioned on sex, age, monthly income, geographical location, urbanization level of the patient’s residence, and year of the index date and adjusted for all other variables.

CI, confidence interval.

## DISCUSSION

In this study, we demonstrated that patients with chronic tonsillitis were more likely to have TC. It is also noteworthy that alcohol abuse/alcohol dependency syndrome was significantly associated with TC. Our findings strongly support tonsillitis and alcohol abuse as potential risk factors for TC.

Traditionally, oropharyngeal cancers share similar pathogenic factors with oral cavity cancers, including alcohol consumption and betel nut chewing [11–13]. The significant association of TC and alcohol abuse found in our study was consistent with these prior studies. In addition, more recently, as the HPV has been more extensively investigated, the role of HPV in the pathogenesis of TC has been widely discussed [14, 15]. Bouda et al. reported that high-risk HPV types are closely associated with oral carcinogenesis [16]. It was also reported that herpes simplex virus might also play a role in oral and oropharyngeal cancers [17, 18]. Some researchers even postulated that high-risk HPV and Epstein-Barr virus co-infections play an important role in initiation of a neoplastic transformation of human oral epithelial cells [19]. While different virus infections do have distinct mechanisms of inducing cancer, what remains unanswered is will inflammation after a latent viral infection actually contribute to viral-induced carcinogenesis?

Inflammatory processes have been linked to carcinogenesis for many years. The theory is not difficult to understand as during inflammatory processes, granulocytes secrete chemically reactive oxidants, radicals, and electrophilic mediators to eradicate pathogens, and these factors are carcinogenic. The best-known example is the association between colon cancer and ulcerative colitis. In a large study done by Ekbom et al. involving a few thousand patients with chronic ulcerative colitis, investigators reported a 5.7-fold increase in the colon cancer incidence compared to a control group [9, 20]. Another study also found that inflammatory bowel disease was correlated with the excess mortality from colorectal cancer [21]. The same phenomenon was observed in the lungs as well. It was reported that patients with asthma showed a significant excess risk of lung cancer [22]. Results from many similar reports also connect the inflammatory process with cancers of the ovaries, pancreas, bladder, skin, and esophagus [9]. Those findings support long-term inflammation eventually elevating the risk of cancer, and carcinogenesis seems to be localized where the inflammation takes place.

It is possible that the development of TC shares common inflammation-induced carcinogenesis mechanisms related to oxidative stress during inflammatory processes. Researchers reported that oxidative stress, oxidants, and antioxidants played a significant role in the pathogenesis of chronic tonsillitis [23, 24]. More interestingly is that there seems to be an interaction between oxidative stress and HPV infection, which is highly associated with TC carcinogenesis as previously mentioned. It was proposed that inflammation generates reactive oxygen species (ROS), which in turn have the potential to create DNA strand breaks, which enable a greater frequency of HPV-DNA integration, and in this way contributes to carcinogenesis [25]. Marco et al. also reported that oxidative stress represents an interesting and under-explored candidate as a promoting factor in HPV-initiated carcinogenesis [26]. They found that HPV16 neoplastic progression seemed to be associated with an increased oxidant environment. Williams et al. demonstrated that the HPV gene product, E6^*^, can further increase ROS levels in host cells, completing this vicious cycle [27]. This phenomenon might partially explain why neoplastic growth is a rare and inappropriate outcome in the natural history of HPV, and many researchers believe that some other events have to concur to induce viral infection into neoplastic transformation [28]. More study is needed to determine if reducing inflammatory processes can contribute to reductions in the TC risk. On the other hand, anti-inflammatory and antioxidative treatments were proven to be effective in reducing infections [29–31]. It would be interesting to know if anti-inflammatory treatments would benefit patients with a higher risk of developing TC.

A limitation of this study, like much research that analyzes health insurance databases, is the possibility of surveillance bias. This means that it is possible that patients with tonsillitis visited doctors more often. It is possible that TC patients with underlying tonsillitis had a better chance of being detected while those in the control group might have remained symptomless and undetected for a certain period. Another limitation is that the LHID2005 used in this study might not include all cases with chronic tonsillitis in Taiwan. Several patients might not receive the ambulatory care since economic reason or with mild symptoms of chronic tonsillitis. Thus, the chronic tonsillitis morbidity might be underestimated in this study.

Moreover, the lack of treatment information for the tonsillitis group is one of the limitations. In this database study, it was difficult to evaluate how these patients were treated. Therefore some patients might have been adequately treated for their tonsillitis or even received a tonsillectomy but were still included in the tonsillitis group. This could potentially have affected the accuracy of our data interpretation. However, if many well-treated tonsillitis subjects were included in the tonsillitis group, the estimated association between chronic tonsillitis and TC might be attenuated. The result would theoretically be affected toward the null hypothesis. Since the results of our study all reached statistically significant differences, we believe this limitation remains fairly acceptable. Additionally, patients’ HPV status, severity of chronic tonsillitis, and levels of inflammatory factors which are lacking in LHID2005 might have had effects on the relationship between chronic tonsillitis and TC.

Finally, some investigators may consider that the underlying demographic characteristics might be unequally distributed between cases and controls at baseline and further affect the relevant findings in this study. For instance, urbanization level and monthly income might affect patients’ intentions to seek medical service. Patients with diverse resident regions might have different lifestyles, diet habits, and prevalence of chronic tonsillitis. However, this study selected the controls by matching some sociodemographics, such as monthly income, geographical location, and urbanization level of the patient’s residence. This methodology could eliminate the demographic difference between two groups.

Through our study, a greater understanding of tonsillitis comorbidities can be achieved. We recommend physicians be more aggressive when dealing with tonsillitis, especially in those groups at high risk for HPV infections, as this common and frequently overlooked disease might potentially contribute to more problems than we expected before.

Despite these limitations, this study found an association between tonsillitis and TC. It is also noteworthy that alcohol abuse/alcohol dependency syndrome was significantly associated with TC (adjusted OR=9.88). We suggest that a replication of the findings is required to further scrutinize and verify the directionality and potential causal link between tonsillitis and TC. We also suggest that clinicians should be aware of this relationship and assess patients with a history of tonsillitis for TC.

## MATERIALS AND METHODS

### Database

We retrieved data for this case-control study from the Longitudinal Health Insurance Database 2005 (LHID2005). The LHID2005, which is derived from medical claims records of the Taiwanese National Health Insurance (NHI) program, comprises original medical claims and registration files for 1,000,000 enrollees under the Taiwanese NHI program. These 1,000,000 enrollees were randomly selected from all enrollees (*n*=23.72 million) listed in the 2005 Registry of Beneficiaries by the Taiwan National Health Institute. The LHID2005, which is available to researchers in Taiwan, provides an exclusive opportunity to explore the association between TC and tonsillitis using a large-scale population-based study.

This study was exempt from full review by the Institutional Review Board of Taipei Medical University (TMU-JIRB N201706061) since the LHID2005 consists of de-identified secondary data released without restriction to researchers for research purposes.

### Study sample

This case-control study included patients with TC (cases) and matched controls. As for cases, we initially identified 714 patients who had received a first-time diagnosis of TC (ICD-9-CM codes 146.0, 146.1, or 146.2) during an ambulatory care visit (including outpatient departments of hospitals and clinics) between January 2001 and December 2013. We excluded five patients under 18 years of age. We then assigned the first claim date with a diagnosis of TC as the index date for cases. We further excluded patients who had a history of a diagnosis of chronic sinusitis (ICD-9-CM codes 473, 473.0, 473.1, 473.2, 473.3, 473.8, and 473.9) (*n*=31) or leukoplakia of the oral mucosa (ICD-9-CM code 528.6) (*n*=50) before the index date. To minimize the bias resulted from the ‘field-cancerization’ effect, we also excluded 139 patients who had received a diagnosis of head and neck cancer (ICD-9-CM codes 140∼148 excluding 146). As a result, 489 patients with TC were included as cases.

We selected five matched controls (*n*=2445) per case from the remaining beneficiaries of the LHID2005. We first assured that none of the selected controls had ever received a diagnosis of TC in any claim. After that, we retrieved the controls matched by sex, age, monthly income (NT$0∼15,840, NT$15,841∼25,000, ≥NT$25,001; the average exchange rate in 2008 was US$1.00≈New Taiwan (NT)$29), geographical location (northern, central, southern, and eastern), urbanization level of the patient’s residence, and year of the index date. Controls were selected by matching them to a given TC case simply on their utilization of medical services in the same index year as that particular case. We assigned the date of the first utilization of ambulatory care occurring in the index year as the index date for controls. Furthermore, we assured that none of the controls had ever received a diagnosis of chronic sinusitis, leukoplakia of the oral mucosa, or head and neck cancer before the index date. Ultimately, 2934 sampled patients were included in this study.

### Exposure assessment

We identified chronic tonsillitis cases based on ICD-9-CM codes 474.0, 474.00, 474.01, and 474.02. We only included those chronic tonsillitis cases who had received at least one diagnosis made by a certified otolaryngologist to increase the validity of the chronic tonsillitis diagnoses. Furthermore, we only included those chronic tonsillitis cases who had received at least one chronic tonsillitis diagnosis within 3 years before the index date.

### Statistical analysis

We used the SAS system for Windows (vers. 8.2, SAS Institute, Cary, NC) to perform all statistical analyses. Chi-squared tests were carried out to explore differences in sociodemographic characteristics as well as medical comorbidities between patients with TC and controls. Medical comorbidities included hypertension, diabetes, hyperlipidemia, obesity, tobacco use disorder, and alcohol abuse/alcohol dependency syndrome. We also used conditional logistic regression analyses (conditioned on sex, age, monthly income, geographical location, urbanization level of the patient’s residence, and year of the index date) to calculate the odds ratio (OR) and corresponding 95% confidence interval (CI) for having been previously diagnosed with chronic tonsillitis between patients with TC and controls. We used the conventional *p*≤0.05 to assess statistical significance.
